# Dysregulated Erythroid Mg^2+^ Efflux in Type 2 Diabetes

**DOI:** 10.3389/fcell.2022.861644

**Published:** 2022-04-04

**Authors:** Ana Ferreira, Alicia Rivera, Jay G. Wohlgemuth, Jeffrey S. Dlott, L. Michael Snyder, Seth L. Alper, Jose R. Romero

**Affiliations:** ^1^ Interdisciplinary Centre of Social Sciences (CICS.NOVA), Faculty of Social Sciences and Humanities (NOVA FCSH), Lisbon, Portugal; ^2^ Department of Medicine, Beth Israel Deaconess Medical Center and Harvard Medical School, Boston, MA, United States; ^3^ Quest Diagnostics, Secaucus, NJ, United States; ^4^ Division of Endocrinology, Diabetes and Hypertension, Department of Medicine, Brigham and Women’s Hospital and Harvard Medical School, Boston, MA, United States

**Keywords:** red blood cells, ion transporter, ion flux, ion exchange, diabetes mellitus, cell magnesium

## Abstract

Hyperglycemia is associated with decreased Mg^2+^ content in red blood cells (RBC), but mechanisms remain unclear. We characterized the regulation of Mg^2+^ efflux by glucose in *ex vivo* human RBC. We observed that hemoglobin A_1C_ (HbA_1C_) values correlated with Na^+^-dependent Mg^2+^ efflux (Na^+^/Mg^2+^ exchange) and inversely correlated with cellular Mg content. Treatment of cells with 50 mM D-glucose, but not with sorbitol, lowered total cellular Mg (2.2 ± 0.1 to 2.0 ± 0.1 mM, *p* < 0.01) and enhanced Na^+^/Mg^2+^ exchange activity [0.60 ± 0.09 to 1.12 ± 0.09 mmol/10^13^ cell × h (flux units, FU), *p* < 0.05]. In contrast, incubation with selective Src family kinase inhibitors PP2 or SU6656 reduced glucose-stimulated exchange activation (*p* < 0.01). Na^+^/Mg^2+^ exchange activity was also higher in RBC from individuals with type 2 diabetes (T2D, 1.19 ± 0.13 FU) than from non-diabetic individuals (0.58 ± 0.05 FU, *p* < 0.01). Increased Na^+^/Mg^2+^ exchange activity in RBC from T2D subjects was associated with lower intracellular Mg content. Similarly increased exchange activity was evident in RBC from the diabetic *db*/*db* mouse model as compared to its non-diabetic control (*p* < 0.03). Extracellular exposure of intact RBC from T2D subjects to recombinant peptidyl-N-glycosidase F (PNGase F) reduced Na^+^/Mg^2+^ exchange activity from 0.98 ± 0.14 to 0.59 ± 0.13 FU (*p* < 0.05) and increased baseline intracellular Mg content (1.8 ± 0.1 mM) to normal values (2.1 ± 0.1 mM, *p* < 0.05). These data suggest that the reduced RBC Mg content of T2D RBC reflects enhanced RBC Na^+^/Mg^2+^ exchange subject to regulation by Src family kinases and by the N-glycosylation state of one or more membrane proteins. The data extend our understanding of dysregulated RBC Mg^2+^ homeostasis in T2D.

## Introduction

Cellular Mg^2+^ levels play a fundamental role in many critical processes, including regulation of cellular ionic composition, enzymatic activity, cell volume and initiation of protein synthesis (Raftos et al., 1999; Rubin, 2005; Vidair and Rubin, 2005). Small variations in cellular or serum Mg content or Mg^2+^ have been associated with various pathological conditions such as diabetes, hypertension, sickle cell anemia and cancer (Sartori et al., 1992; Resnick et al., 1993a; Picado et al., 1994; Barbagallo et al., 1996; Dewitte et al., 2004; Maltezos et al., 2004; Zehtabchi et al., 2004). Mammalian cells maintain cellular Mg^2+^ levels within a narrow range by the combined action of ion transporters and cellular Mg^2+^ buffering capacity. ATP, 2,3-bisphosphoglycerate, cellular phosphoproteins (including phosphorylation-regulated protein kinases and phosphatases themselves) and other nucleic acids (including polynucleic acids) constitute the major cellular Mg^2+^ buffers (Laing et al., 1994; Raftos et al., 1999; Chiu and Dickerson, 2000; Waas and Dalby, 2003). Cellular Mg^2+^ transport encompasses both influx of extracellular Mg^2+^, attributed to TRPM6/7 channels and (more controversially) to CNNM and MagT1 transporters (Nadler et al., 2001; Runnels et al., 2001; Monteilh-Zoller et al., 2003; Schmitz et al., 2003; Voets et al., 2004; Goytain and Quamme, 2008; Giménez-Mascarell et al., 2019), and efflux of intracellular Mg^2+^, mediated by both Na^+^-dependent and Na^+^-independent mechanisms (Féray and Garay, 1986; Ferreira et al., 2004). Na^+^-dependent Mg^2+^ efflux (Na^+^/Mg^2+^ exchange) has been functionally described in various cell types including human RBC (Féray and Garay, 1986; Murphy et al., 1991; Günther and Vormann, 1992; Romani et al., 1993; Wolf et al., 1997; Touyz and Schiffrin, 1999a; Touyz et al., 2001; Standley and Standley, 2002; Ferreira et al., 2004). We and others have shown that Mg^2+^ transport across the RBC plasma membrane is regulated by insulin, norepinephrine, angiotensin II, and vasopressin through activation of Na^+^/Mg^2+^ exchange (Romani et al., 1993; Touyz and Schiffrin, 1999b; Cefaratti and Romani, 2003; Ferreira et al., 2004; He et al., 2005; Rubin, 2005). The *SLC41A1* gene has been proposed to encode the erythroid Na^+^/Mg^2+^ exchanger polypeptide (Schweigel et al., 2000; Sahni et al., 2007; Kolisek et al., 2008; Kolisek et al., 2012), a hypothesis strengthened by the demonstration that SLC41A3 mediates mitochondrial Na^+^/Mg^2+^ exchange (Mastrototaro et al., 2016). However, more a more recent report has failed to detect extracellular Na^+^-dependence of SLC41A1-mediated Mg^2+^ transport (Arjona et al., 2019).

Type 2 diabetes (T2D) affects nearly 500 million people worldwide, with a rapidly rising annual toll exceeding four million deaths (Palomino-Schätzlein et al., 2020). The multiple pathophysiological effects of hyperglycemia are not completely explained by glucose-induced changes in cellular energy stores or in red cell (Morabito et al., 2020) and mononuclear cell oxidative stress (Fortuño et al., 2006). In particular, the relationship between divalent cation metabolism and the pathogenesis and complications of hyperglycemic, often insulin-resistant conditions such as T2D is not fully understood. The hypomagnesemia observed in up to half of people with T2D (Gommers et al., 2016), as well as in many children with insulin-dependent diabetes (Rohn et al., 1993), has been traditionally attributed to urinary magnesium wasting secondary to the osmotic diuresis accompanying diabetic glycosuria (Gommers et al., 2016). In contrast, elevated glucose concentrations have been shown to lower RBC Mg content through as yet undefined mechanisms (Paolisso et al., 1986; Resnick et al., 1993b; John, 1997; Rodríguez-Morán et al., 2011; Chan et al., 2015). Serum glucose concentrations are associated with low Mg^2+^ levels in both serum and RBC (Resnick et al., 1993b; Guerrero-Romero and Rodríguez-Morán, 2002; Rodríguez-Morán and Guerrero-Romero, 2003). The mechanisms by which glucose induces reduction in cellular Mg content and contributes to development of diabetic complication are not completely understood. Elevated serum glucose levels lead to glycation of circulating and of membrane-bound proteins. Indeed, glycated HbA_1c_ is an excellent clinical marker of glycemic status over time (John, 1997; Pani et al., 2008). HbA_1c_ in T2D subjects is higher than in control subjects and negatively correlates with Mg levels in plasma, platelets, mononuclear cells, and muscle cells (Allegra et al., 1997).

We now present evidence that glucose modulates erythrocyte Mg levels by stimulating Mg^2+^ efflux through activation of Na^+^/Mg^2+^ exchange in *ex vivo* RBC from humans with type 2 diabetes (T2D) and otherwise healthy individuals, as well as in a rodent model of T2D. We also show that RBC from T2D subjects have lower cellular Mg content and increased baseline Na^+^/Mg^2+^ exchange activity as compared to cells from normal control subjects. Treatment of RBC from T2D subjects with extracellular N-glycosidase increased cellular Mg content and decreased Na^+^/Mg^2+^ exchange activity to non-diabetic control levels. Thus, our results suggest that RBC protein glycation adducts and protein N-glycans directly or indirectly modulate Na^+^/Mg^2+^ exchanger activity, contributing to dysregulation of Mg^2+^ homeostasis in T2D.

## Experimental Procedures

*Materials:* A23187, bovine serum albumin (BSA, fraction V), choline chloride (Choline Cl), PP2 and SU6656 were purchased from Calbiochem (San Diego, CA). Wortmannin (WT) was from Alexis Corp. (San Diego, CA). Dimethyl sulfoxide (DMSO) was from Fisher Scientific (Pittsburgh, PA). Acationox was from Baxter Scientific Products (McGaw Park, IL). All other reagents were purchased from Sigma Aldrich (St. Louis, MO).

*Isolation of Red Cells:* Human blood samples were obtained between 7:00 a.m. and 10:00 a.m. after overnight fast (>6 h) per approved clinical protocol and processed within 3 h of receipt, as previously described (Ferreira et al., 2004; Rivera et al., 2005). Briefly, freshly isolated blood was passed through cotton and eluted with choline wash solution (CWS-Mg free, containing (in mM) 150 choline chloride, 10 Tris MOPS pH 7.4 at 4°C) to remove the buffy coat (>85% of white blood cells and platelets). The red cells were washed 4× at 4°C with CWS-Mg free kept on ice until use. Hematological parameters were measured by ADVIA hemoanalyzer (Bayer, Terrytown, NY). Glycated hemoglobin (HbA_1c_) was measured by Hitachi 917 autoanalyzer (Manheim/Boehringer). Total intracellular contents of Na, K, and Mg were measured by atomic absorption spectrophotometry (Perkin Elmer 800). To avoid changes in mean cellular volumes (MCV), mouse blood was prepared as specified for human RBC, but in solutions adjusted to the osmolarity of normal mouse plasma (330 mOsm).

*Cellular Mg* ^*2+*^ *efflux measurements:* Cells were loaded with Mg^2+^ as we previously described (Ferreira et al., 2004; Rivera et al., 2005). Cells at 10% hematocrit were incubated with 6 µM Ca^2+^/Mg^2+^ ionophore A23187 in Mg^2+^-loading solution (MLS) containing (in mM) 140 KCl, 12 MgCl_2_, 10 D-glucose, and 10 Tris MOPS (pH 7.4) for 30 min at 37°C. To obtain a range of different intracellular Mg concentrations in Figure 5B, MLS solutions contained 0–16 mM MgCl_2_ concentrations between 0 and 16 mM, with corresponding KCl concentrations between 138 and 158 mM. A23187 was subsequently removed with four washes of 25 volumes of 0.1% BSA in MLS at 37°C at 15 min intervals. Mg^2+^-loaded cells at 3% hematocrit were incubated at 37°C for periods of 5 or 45 min in NaCl or choline choline chloride flux media containing (in mM) 140 NaCl or 140 choline Cl, 10 D-glucose, 10 Tris MOPS (pH 7.4), 20 sucrose, 0.1 ouabain, and 0.01 bumetanide. Mg^2+^ efflux was calculated from the slope of linear regression analyses of Mg content (expressed as calculated concentration) in supernatant vs. time (5 and 45 min in triplicate determinations). Na^+^/Mg^2+^ exchange activity was calculated as the difference between Mg^2+^ efflux in NaCl and in choline Cl flux media. Flux values were corrected for changes in mean corpuscular volume (MCV) and expressed as mmol/10^13^ cells × h (flux units, FU), as previously described (Ferreira et al., 2004; Rivera et al., 2005). Mouse RBC assay osmolytes were adjusted to reflect the normal mouse plasma osmolarity of ∼330 mOsm, to minimize cell volume changes and hemolysis during Mg^2+^ loading (Rivera et al., 2005).

*Glucose-induced Mg* ^*2+*^ *efflux*: RBC were incubated up to 24 h at 37°C in isotonic saline solution containing (in mM): 140 NaCl, 0–100 D-glucose, 10 Tris MOPS (pH 7.4), 0–20 sucrose, 0.1 ouabain, and 0.01 bumetanide, with the indicated concentrations of D-glucose or sorbitol. Incubated RBC were centrifuged 5 min at 2,500 rpm at 37°C and suspended in Mg^2+^-free choline wash solution as previously described (Acosta et al., 2000). Previous investigation (Viskupicova et al., 2015) revealed that 1 h exposure of RBC to >45 mM glucose at 37°C does not increase hemolysis, eryptosis or GSSG/GSH ratio (Viskupicova et al., 2015). Moreover, lipid peroxidation, superoxide production and intracellular (Ca^2+^) remain unchanged under these conditions. We observed no hemolysis after 24 h incubation of RBC in the presence of 50 mM glucose. We noted trace hemolysis after 24 h incubation in the presence of 100 mM glucose, but no further hemolysis was evident in RBC after resuspension in flux medium. Aliquots of 50% suspension were taken for measurements of Mg^2+^ efflux and total cellular contents of Mg, K, and Na as described above. We also studied the effects of Peptidyl-N-Glycosidase F (PNGase F), an amidase that cleaves the GlcNAc-asparagine linkage of high mannose and complex oligosaccharides of N-linked glycoproteins (Maley et al., 1986). Intact RBC were incubated with 0.1 U/ml PNGase F (New England BioLabs, Ipswich, MA) for 1.5 h at 37°C in isotonic saline solution, then centrifuged 5 min at 2,500 rpm at 37°C and resuspended in Mg^2+^-free choline wash solution as described (Tarentino and Plummer, 1994). Timed aliquots of 50% suspension were taken for measurement of total RBC Mg, K, and Na.

*Study subjects:* The study protocol was approved by the Institutional Review Board of the Brigham and Women’s Hospital (Protocol #: 2003P001861). Normal (*n* = 33) and T2D individuals (*n* = 30) were recruited at the Brigham and Women’s Hospital. Informed written consent was obtained from all subjects before participation. T2D was diagnosed according to accepted guidelines (National Diabetes Data Group: Classification and diagnosis of diabetes mellitus and other categories of glucose intolerance, 1979). Diabetic participants were not being treated with antihypertensive or any other medication except for medication related to their diabetes. Not all subjects were studied in all analyses, and subjects were not matched for clinical characteristics. Subjects varied in age between 22 and 62, with body mass index (BMI) <31 kg/m^2^ for women or <33 kg/m^2^ for men. Diabetic and control groups did not differ significantly in age.

*db/db Mice:* All mouse studies were conducted under protocols approved by the Institutional Animal Care and Use Committee of Brigham and Women’s Hospital. *db/db* mice (The Jackson Laboratory; Bar Harbor, Maine; Catalog #000642) and heterozygote controls (Catalog #000662) were purchased at 6 weeks of age, fed *ad libitum* with rodent chow (LabDiet #5053; Richmond, IN) and water, then sacrificed and venisected by intracardiac puncture at 25 weeks of age. *db/db* mice are homozygous for a spontaneous mutation of the leptin receptor (*Lepr* ^*db*^) leading to unrestrained weight gain and eventual development of obesity-associated diabetes. *db/db* but not *db/+* mice exhibit hyperglycemia as early as 8 weeks of age and have been used to model human T2D (Shafrir and Sima, 2001; Joost and Schürmann, 2014). Although not measured in these experimental mouse groups, blood glucose levels in mice of the same age (25 weeks) from the same supplier and maintained in the same animal facility had blood glucose levels of 159 ± 21 mg/dl (*db/+*) and 769 ± 41 mg/dl (*db/db*) (Guo et al., 2008).

*Statistical Analyses*: The data are reported as means ± standard error of the mean (SEM), with statistical significance (*p* value as indicated) determined by non-parametric analysis t-test unless otherwise stated. The kinetic parameters of Na^+^/Mg^2+^ exchange activation were analyzed with GraphPad Prism version 8.0.0 for Windows (GraphPad Software, San Diego, California United States). Pearson and Spearman coefficients of correlation were estimated using SPSS 10.0 software (SPSS, Chicago, IL).

## Results

### Glycated Hemoglobin (HbA_1C_) Correlates With Cellular Mg^2+^ and Na^+^/Mg^2+^ Exchange Activity

**FIGURE 1:**
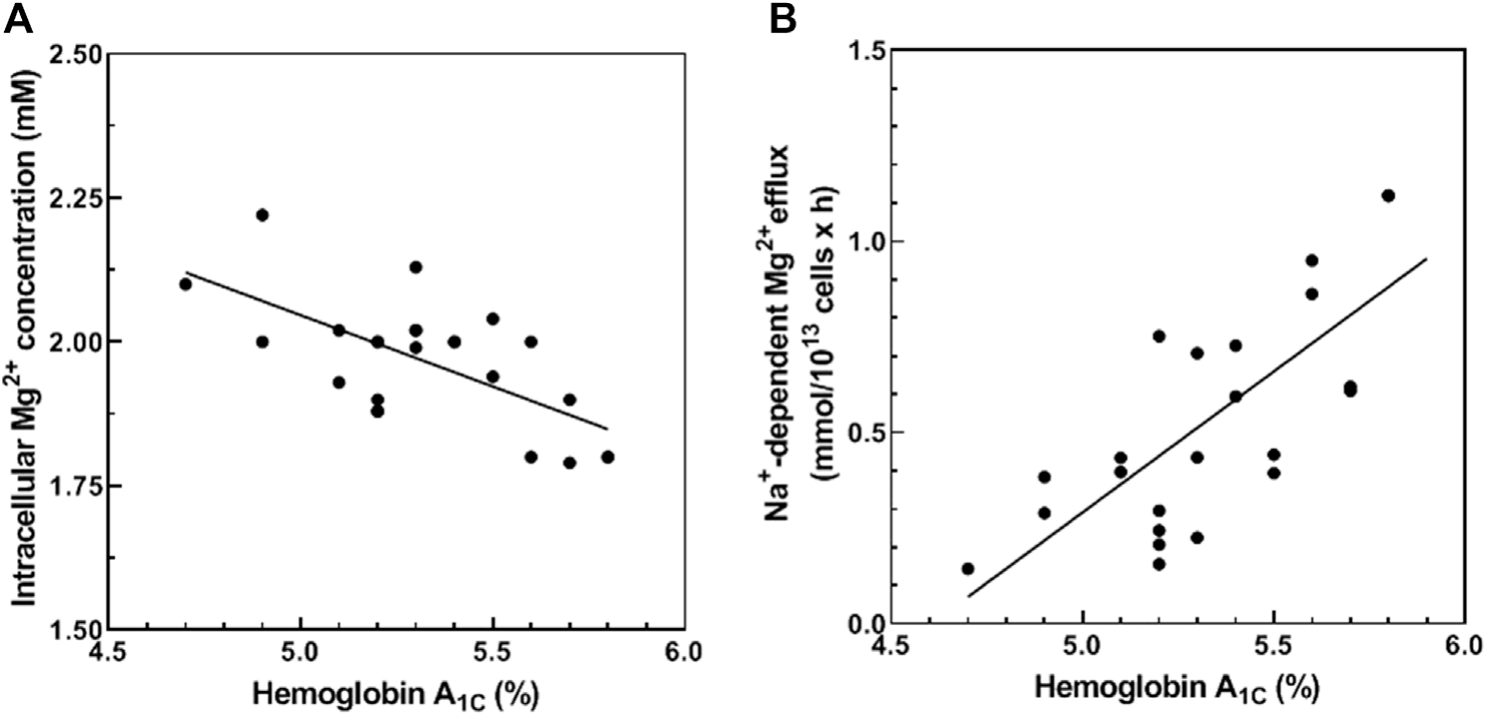
Correlation between HbA_1C_ and total cellular Mg^2+^ content **(A)** and Na^+^-dependent Mg^2+^ efflux. **(A)** Cells from 23 normal subjects were assayed for hemoglobin A_1C_ (HbA_1C_) and total cellular Mg^2+^ content as described in Methods. The graph represents total cellular Mg^2+^ content vs. HbA_1C_ (*n* = 23, r = −0.600 and *p* < 0.01; *σ* = −0.491 and *p* < 0.05). **(B)** Cells from 23 normal subjects were assayed for HbA_1C_ and Na^+^-dependent Mg^2+^ efflux (Na^+^/Mg^2+^ exchanger activity) as described in Methods. The graph represents Na^+^/Mg^2+^ exchanger activity vs. HbA_1C_ (*n* = 23, *p* < 0.001 and r = 0.716 and *p* < 0.001; *σ* = 695 and *p* < 0.001).

We hypothesized that RBC membrane protein glycation (or another consequence of chronic hyperglycemia) may alter Mg^2+^ homeostasis. To investigate the relationship between glycemic status and red cell Mg^2+^ homeostasis, we examined the correlation between HbA_1c_ and erythrocyte Mg^2+^ levels in normal red cells. We observed a negative correlation between baseline levels of total cellular Mg^2+^ content and HbA_1C_ levels (Pearson r = −0.600, *p* < 0.01; Spearman *σ* = −0.491 and *p* < 0.05, *n* = 23) (Figure 1A). These results suggest a relationship between RBC glycation state and increased Mg^2+^ loss from RBC.

Cellular Mg^2+^ transport across the plasma membrane is mediated by Na^+^-dependent and Na^+^-independent mechanisms. We and others have shown that Na^+^-dependent Mg^2+^ efflux (Na^+^/Mg^2+^ exchange activity) contributes to RBC Mg^2+^ homeostasis (Féray and Garay, 1986; Touyz et al., 2001; Ferreira et al., 2004). In this study, we measured HbA_1C_ levels and Na^+^/Mg^2+^ exchange activity in RBC from normal subjects. Normal RBC revealed a positive correlation between HbA_1C_ and Na^+^/Mg^2+^ exchange activity (Pearson r = 0.716, *p* < 0.001; Spearman *σ* = 0.695, *p* < 0.001, *n* = 23) (Figure 1B). These results are consistent with a regulatory mechanism linking Mg^2+^ homeostasis and cell glycemic status. In contrast, HbA_1C_ levels correlated poorly with Na^+^-independent Mg^2+^ efflux activity of normal RBC (Pearson r = 0.142, *p* = 0.270; Spearman *σ* = 0.225, *p* = 0.163, *n* = 23, data not shown). These data suggest that reduced Mg content of RBC in hyperglycemic individuals reflects, at least in part, modulation of Na^+^/Mg^2+^ exchange activity.

### Effect of Glucose on Red Blood Cells Mg^2+^ Levels and Na^+^/Mg^2+^ Exchanger Activity *In Vitro*

**FIGURE 2:**
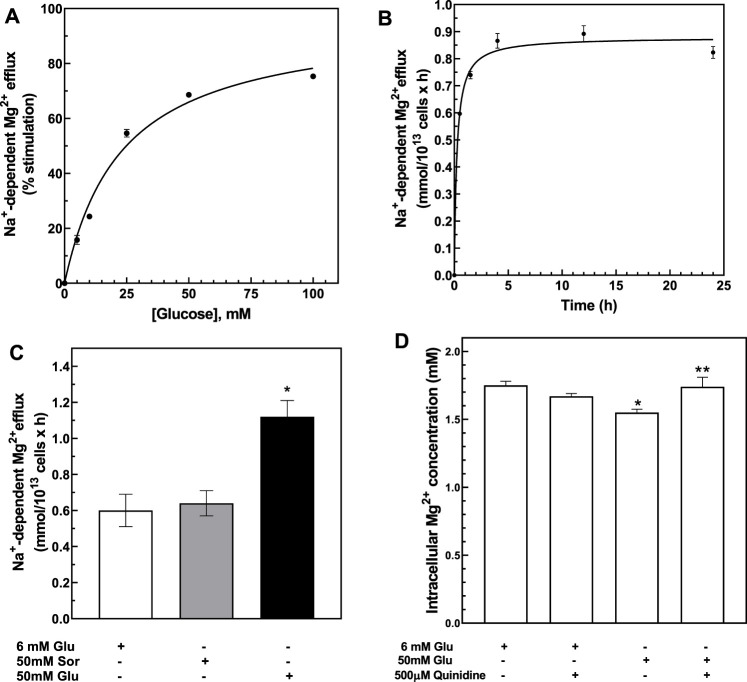
Activation of Na^+^-dependent Mg^2+^ efflux by glucose. **(A)** Glucose dose-dependent activation of Na^+^-dependent Mg^2+^ efflux. Cells from normal subjects were incubated with 0–100 mM D-glucose for 24 h and assayed for Na^+^-dependent Mg^2+^ efflux or Na^+^/Mg^2+^ exchanger activity. Hyperbolic pattern curve best fit was used to derive V_max_ = 0.978 ± 0.01 FU, K_m_ = 3.2 ± 0.5 mM. Plot shows % of stimulation over 0 glucose present. Values are expressed as means ± SE of three experiments in triplicate determinations (r = 0.998). Plot shows % of stimulation over 0 glucose present. **(B)** Time course of glucose-dependent activation of the Na^+^/Mg^2+^ exchanger. Cells from normal subjects were incubated with 50 mM D-glucose for 0–24 h and assayed for Na^+^/Mg^2+^ exchanger activity as described in Methods. Hyperbolic pattern curve best fit was used to derive V_max_ = 0.98 ± 0.01 FU, reaching half-activity in 1.6 ± 0.0 h (r = 0.90). Values are expressed as means ± SEM of three experiments in triplicate determinations. **(C)** Activation of Na^+^/Mg^2+^ exchanger by glucose but not sorbitol. Cells from normal subjects were incubated with 6 or 50 mM D-glucose or 50 mM sorbitol solutions for 24 h and assayed for Na^+^/Mg^2+^ exchanger activity as described in Methods. Values represent the activity of the exchanger and are expressed as means ± SEM. (* paired non-parametric *t* test, *p* < 0.005, *n* = 3). **(D)** Effect of quinidine on glucose-induced cellular Mg^2+^ levels. Cells from normal subjects were incubated with 6 or 50 mM glucose and in the presence or absence of 500 μM quinidine for 1.5 h and assayed for total cellular Mg^2+^ content as described in Methods. Results are expressed as means ± SEM of four experiment in triplicates. (* unpaired non-parametric t test 6 vs. 50 mM glucose *p* < 0.05; ** paired non-parametric t test 50 mM glucose vs. 50 mM glucose plus 500 μM quinidine *p* < 0.04).

To study the mechanisms involved in RBC Mg^2+^ regulation by high glycemic status, we assayed Na^+^/Mg^2+^ exchange activity as a function of extracellular glucose concentration from 0 to 100 mM, as described in Methods. RBC exposure to up to 100 mM glucose for 24 h at 37°C is known not to increase hemolysis, eryptosis, or concentrations of HbA_1C_ or intracellular Ca^2+^ (Viskupicova et al., 2015). We observed that Mg^2+^-loaded RBC exhibited a glucose concentration-dependent increase in Na^+^/Mg^2+^ exchange activity (Figure 2A). Kinetic analyses of the hyperbolic curve (r = 0.998) indicated a maximal velocity (V_max_) of 0.978 ± 0.01 mmol/10^13^ cell × h and EC_50_ of 18.1 ± 3.0 mM, representing a ∼50% increase over basal exchange activity in the presence of 100 mM glucose. In contrast, Na^+^-independent Mg^2+^ efflux was unaffected by changing extracellular glucose concentration (r = 0.620, data not shown). These results show that exposure of erythrocytes to high glucose media will modify cellular Mg^2+^ homeostasis.

To characterize the glucose-mediated activation of Na^+^/Mg^2+^ exchange, we monitored time-dependent changes in exchange activity. RBC stimulated by 50 mM D-glucose were examined for Na^+^/Mg^2+^ exchange activity at 0.5, 1.5, 4, 12, and 24 h. Significant change in Na^+^/Mg^2+^ exchange activity was detected after 1.5 h, with peak response observed as early as 4 h (Figure 2B). We also measured Na^+^/Mg^2+^ exchange activity at baseline conditions in Mg^2+^-unloaded RBC after 4 h exposure to either 6 mM or 50 mM D-glucose. RBC incubation with 50 mM glucose increased Na^+^/Mg^2+^ exchange activity from 0.006 ± 0.003 to 0.013 ± 0.005 mmol/10^13^ cells × h (*n* = 3, *p* < 0.05). Thus, high glucose stimulates Na^+^/Mg^2+^ exchange activity in both Mg^2+^-loaded and -unloaded cells.

We hypothesized that altering the glycation state of RBC surface proteins may change intracellular Mg^2+^ homeostasis. Protein glycation rate in RBC is time- and glucose concentration-dependent (Watala, 1988). At high glucose concentration, most membrane protein glycation occurs within 12 h (Watala, 1988). To examine whether glycation status would affect the exchanger activity, we first examined the effects on Na^+^/Mg^2+^ exchange activity of the non-glycating sugar, sorbitol (Yan et al., 2003), and compared its effects to those of glucose. RBC were incubated with physiological concentrations of either D-glucose (6 mM), high D-glucose (50 mM) or 50 mM sorbitol for 24 h at 37°C. As previously observed, exchange activity was higher in RBC incubated with 50 mM D-glucose than in RBC incubated either at the physiological glucose level of 6 mM or in 50 mM sorbitol (*n* = 3, *p* < 0.05) (Figure 2C). These results are consistent with the possibility that RBC membrane glycation state alters Na^+^/Mg^2+^ exchange activity. We also observed a significant decrease in total cell Mg content in RBC incubated with 50 mM glucose (1.55 ± 0.02 mM) as compared to cells incubated with 6 mM glucose (1.75 ± 0.03 mM, *n* = 3, *p* < 0.01). In contrast, incubation of cells with 50 mM sorbitol failed to alter RBC Mg levels (1.8 ± 0.02 mM, *n* = 3, data not shown). These observations suggest that the possibility that increased RBC surface glycation might stimulate Na^+^/Mg^2+^ exchange.

We and others have reported that quinidine inhibits red cell Na^+^/Mg^2+^ exchange (Féray and Garay, 1986; Murphy et al., 1991; Günther and Vormann, 1992; Picado et al., 1994; Touyz and Schiffrin, 1999a; Touyz et al., 2001; Günther, 2006). We tested the effect of quinidine on glucose-induced loss of intracellular Mg from RBC. Quinidine (500 microM) prevented the intracellular loss of Mg^2+^ induced by 50 mM D-glucose (Figure 2D). These observations are consistent with a role for Na^+^/Mg^2+^ exchange in mediating glucose-induced Mg^2+^ loss from RBC.

### Cellular Mg^2+^ Regulation in Red Blood Cells From Type 2 Diabetic Subjects

Our model predicts that RBC from T2D subjects should exhibit increased Na^+^/Mg^2+^ exchange activity. We first examined various red cell parameters in freshly isolated RBC from otherwise healthy normal and T2D subjects. We found no significant differences in total cellular Na and K content, mean corpuscular volume (MCV) or mean corpuscular hemoglobin concentration (MCHC). However, total cellular Mg^2+^ levels were significantly lower in RBC from T2D subjects than in those from control subjects (Table 1), as previously reported (Djurhuus et al., 1995; Eibl et al., 1995; Vidair and Rubin, 2005).

**TABLE 1:** Hematological values of normal and diabetic subjects.

	Normal	Diabetic	*p* value
N	20	16	—
MCV, %	91.7 ± 1.3	94.3 ± 1.0	0.700
MCHC, g/dL	32.7 ± 0.4	32.4 ± 0.4	0.700
Reticulocytes, %	1.9 ± 0.1	1.6 ± 0.2	0.522
RDW	14.1 ± 0.2	14.0 ± 0.3	0.621
Na^+^, mM	8.3 ± 0.8	6.9 ± 0.6	0.538
K^+^, mM	84.3 ± 3.4	86.2 ± 2.6	0.700
Mg^2+^, mM	2.3 ± 0.1	2.0 ± 0.1	<0.001

Mean cellular volume (MCV); mean corpuscular hemoglobin concentration (MCHC); red cell density width (RDW); total cellular Na^+^; K^+^ and Mg^2+^ were determined as described in Methods. *p* values were determined by unpaired Mann-Whitney test.

**FIGURE 3:**
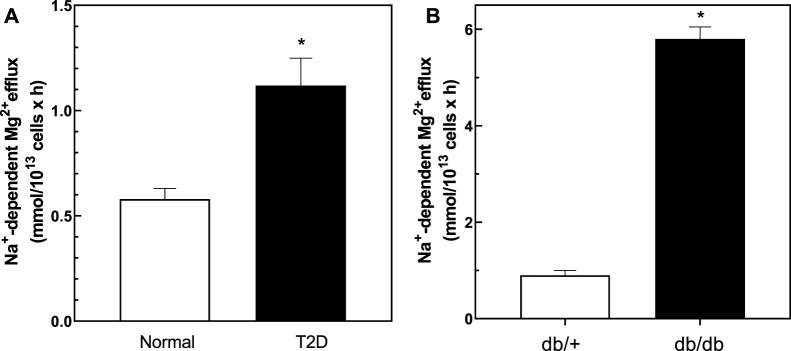
Baseline Na^+^/Mg^2+^ exchanger activity in cells from normal and type 2 diabetic human subjects and db/db and db/+ mice. Cells were used to determine the activity of the Na^+^/Mg^2+^ exchanger as described in Methods. **(A)** Na^+^/Mg^2+^ exchanger activity in cells from normal and T2D human subjects (*n* = 33 and *n* = 30 in normal and diabetic subjects, respectively; * unpaired t test, *p* < 0.001). **(B)** Na^+^/Mg^2+^ exchanger activity in red cells from diabetic db/db and non-diabetic db/+ mice (*n* = 3); * unpaired *t* test nonparametric, *p* < 0.01). Results are expressed as means ± SEM of three experiments in triplicate determinations.

We measured Na^+^/Mg^2+^ exchange activity in T2D and control RBC. We found that Na^+^/Mg^2+^ exchange activity was higher in T2D RBC (1.19 ± 0.13 FU; *n* = 33) than in RBC from normal subjects (0.58 ± 0.05 FU; *n* = 30, *p* = 0.0011) (Figure 3A). In contrast, Na^+^-independent Mg^2+^ efflux from T2D RBC (3.57 ± 0.53 FU; *n* = 30) did not differ statistically from that of normal RBC (2.51 ± 0.37 FU, *n* = 30; means ± SEM). These data are consistent with a major role for Na^+^/Mg^2+^ exchange in Mg^2+^ loss from T2D RBC. Furthermore, exposure to quinidine (500 μM) decreased Na^+^/Mg^2+^ exchange activity in RBC from T2D subjects by 0.44 ± 0.06 FU (*n* = 3) but only by 0.18 ± 0.07 FU (*n* = 4) in normal RBC (data not shown).

We also examined cellular Mg^2+^ levels and exchanger activity in *ex vivo* RBC from *db/db* mice, a well-described model of T2D, and in RBC from non-diabetic *db/+* mice (Figure 3B). Consistent with our observations in T2D subjects, RBC from 25 weeks old *db/db* mice exhibited lower cellular Mg^2+^ content (2.3 ± 0.2 mmol/kg Hb) than RBC of *db/+* control mice of similar age (4.4 ± 0.5, *n* = 3; *p* < 0.03). *db/db* diabetic mouse RBC also exhibited higher rates of Na^+^/Mg^2+^ exchange activity than did RBC of nondiabetic *db/+* mice (Figure 3B).

### Src-Kinase Inhibitors Regulate Glucose-Stimulated Na^+^/Mg^2+^ Exchanger Activity

**FIGURE 4:**
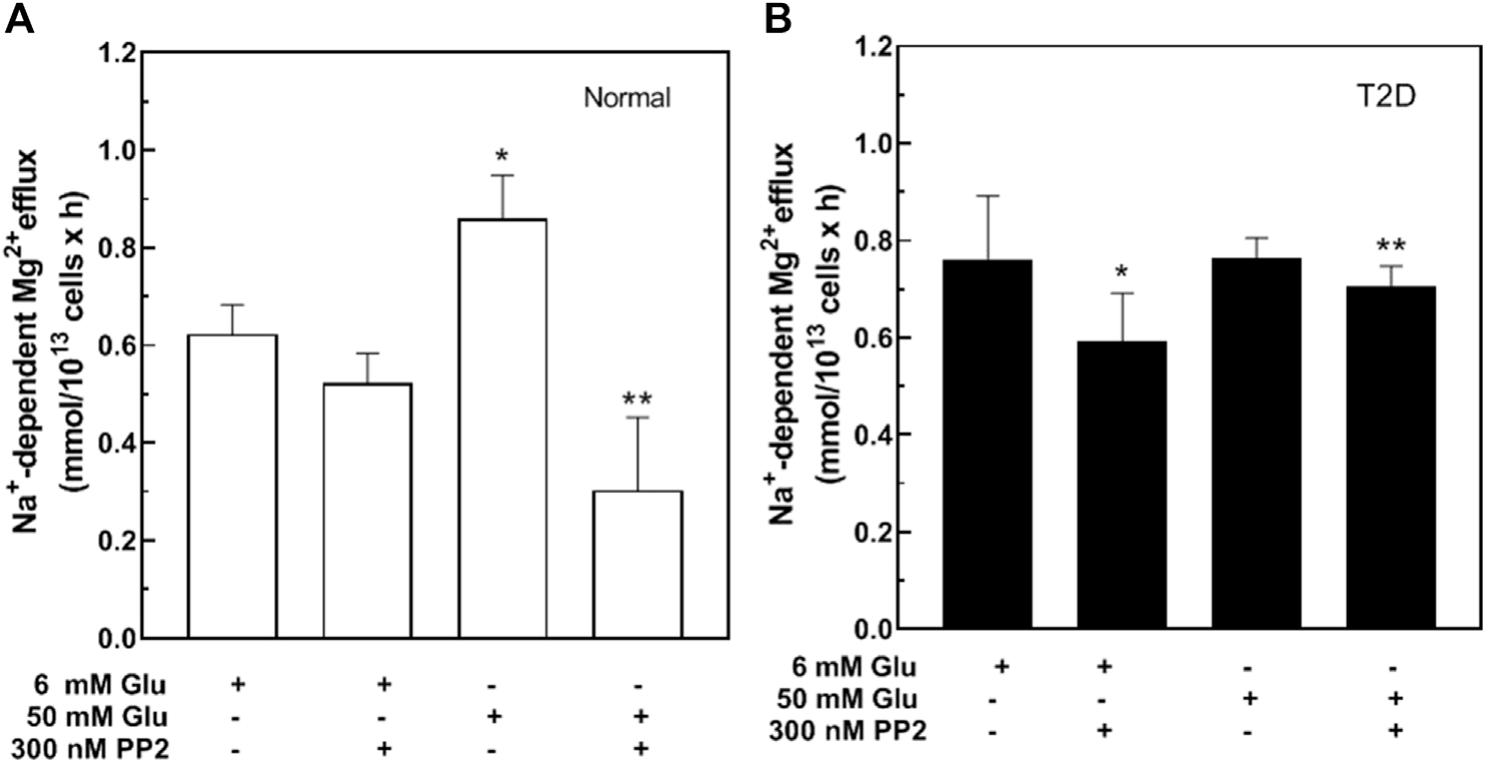
Effect of Src inhibitors on Na^+^/Mg^2+^ exchanger activity in cells from type 2 diabetic subjects. Cells were incubated in glucose solutions of 6 mM (106 mg/dl) or 50 mM (900 mg/dl), in the presence or absence of 300 nM PP2 as indicated, then assayed for Na^+^/Mg^2+^ exchanger activity. **(A)** Values represent exchanger activity in cells from normal subjects and are expressed as means ± SEM of three experiments in triplicate determinations. Non-parametric paired t test, *p < 0.003 **p < 0.002 from basal activity of the exchanger; **(B)** Values represent exchanger activity in cells from diabetic subjects and are expressed as means ± SEM of three experiments in triplicates. Non-parametric paired t test *p < 0.05, **p < 0.05 from basal activity.

Hyperglycemia increases Src family tyrosine kinase activity that in turn increases production of reactive oxygen species (Schaeffer et al., 2003). To evaluate the role of Src kinases on Na^+^/Mg^2+^ exchanger activity, normal RBC were treated with either 6 or 50 mM glucose in the presence or absence of the Src inhibitor, PP2 (Figure 4A). Incubation with PP2 did not alter Na^+^/Mg^2+^ exchanger activity in the absence of glucose, but blocked the glucose-induced increase of exchanger activity. Similar results were observed in normal RBC using a structurally dissimilar Src inhibitor, SU6656 (300 nM) (*n* = 3, *p* < 0.01), and known “off-target” effects of SU6656 (Gao et al., 2013; Ross et al., 2017) and PP2 (Brandvold et al., 2012) do not overlap among red cell kinases or those of other cells. We also tested the effects of Src inhibitors in RBC from T2D subjects, under similar conditions. PP2 inhibited exchanger activity in T2D RBC stimulated either by 6 or 50 mM D-glucose (Figure 4B), suggesting that a Src kinase-regulated pathway mediates the glucose-induced increase in RBC Na^+^/Mg^2+^ exchange activity.

### Na^+^/Mg^2+^ Exchange Activity as a Function of Extracellular Na^+^ and Intracellular Mg^2+^

**FIGURE 5:**
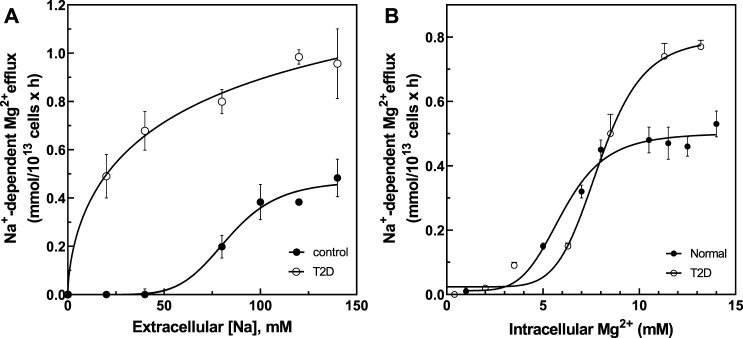
Activation of Na^+^/Mg^2+^ exchanger by extracellular Na^+^ and intracellular Mg^2+^ in cells from normal and type 2 diabetic subjects. **(A)** Activation of Na^+^/Mg^2+^ exchanger by extracellular Na^+^ in normal (closed circles, V_max_ = 0.472 ± 0.01 FU, hill = 6.4 ± 0.13, K_1/2_ = 83.5 mM, *n* = 3) and diabetic subjects (open circles, V_max_ = 1.16 ± 0.05 FU, K_m_ = 28.9 ± mM, *n* = 3). **(B)** Activation of Na^+^/Mg^2+^ exchanger by intracellular Mg^2+^ in normal (closed circles, V_max_ = 0.50 ± 0.02 mmol/10^13^ cell × h, hill = 5.1 ± 0.1, K_1/2_ = 5.97 ± 0.2 mM, *n* = 3) and diabetic subjects (open circles, V_max_ = 0.81 ± 0.032 FU, hill = 6.3 ± 0.2, K_1/2_ = 7.87 ± 0.39 mM, *n* = 3). Values represent the activity of the Na^+^/Mg^2+^ exchanger and are expressed as means ± SEM of *n* = 3 in triplicates determinations.

We previously reported that V_max_ of Na^+^/Mg^2+^ exchange activity in RBC from patients with sickle cell disease (HbSS) was higher than in cells from subjects with normal hemoglobin A expression (HbAA) (Rivera et al., 2005) due in part to changes in extracellular Na^+^ affinity and not through changes in intracellular Mg^2+^ affinity. These results suggested possible cooperativity of extracellular Na^+^ binding to the exchanger at basal state. To investigate whether similar mechanisms might apply to Na^+^/Mg^2+^ exchange in T2D RBC, we assayed Na^+^/Mg^2+^ exchange activity in RBC from normal and T2D subjects as a function of extracellular (Na^+^) (Figure 5A). In T2D RBC the plot of exchange activity vs. extracellular (Na^+^) was best fit by a hyperbolic Michaelis-Menten curve (r = 0.995) exhibiting a K_m_ of 28.9 ± 2.4 mM for external Na^+^ and a V_max_ of 1.16 ± 0.05 FU (*n* = 3, r = 0.99). In contrast, the Na^+^ -dependence of Na^+^/Mg^2+^ exchange activity in normal RBC was best fit by a sigmoidal curve (n = 3, r = 0.99) with an affinity constant for external Na^+^ of 83.5 ± 4 mM (*p* < 0.0001 vs. T2D) and a V_max_ of 0.472 ± 0.01 FU, (*p* < 0.0001 vs. T2D), with Hill coefficient (n) of 6.4 ± 0.13. These results suggest that increased Na^+^/Mg^2+^ exchanger activity in T2D subjects may reflect increased affinity at extracellular Na^+^ binding sites possibly attributable to chronic exposure to high glucose.

The affinity for intracellular Mg^2+^ was also examined (Figure 5B). Cells were Mg-loaded to estimated intracellular concentrations between ∼0 and ∼14 mM (Figure 5B). Increasing intracellular Mg^2+^ stimulated exchanger activity in a sigmoidal pattern in RBC of both normal (r = 0.993) and T2D subjects (r = 0.996). RBC from normal subjects exhibited an affinity constant for intracellular Mg^2+^ of 5.97 ± 0.2 mM with Hill coefficient of 5.1 ± 0.1, as compared to an affinity constant of 7.87 ± 0.39 mM (n.s.) with Hill coefficient of 6.3 ± 0.2 in T2D red cells (both *n* = 3). V_max_ in RBC from T2D subjects was 0.81 ± 0.032 FU vs. 0.50 ± 0.02 FU in RBC from normal subjects (*p* = 0.003). Since the affinity constants for intracellular Mg^2+^ were statistically indistinguishable in T2D and normal RBC, we propose that changes in external Na^+^ binding affinity and V_max_ drive increased Na^+^/Mg^2+^ exchange activity in T2D RBC.

### Regulation of Mg^2+^ Levels and Na^+^/Mg^2+^ Exchanger Activity *In Vitro* by Pre-Treatment With PNGase F

**FIGURE 6:**
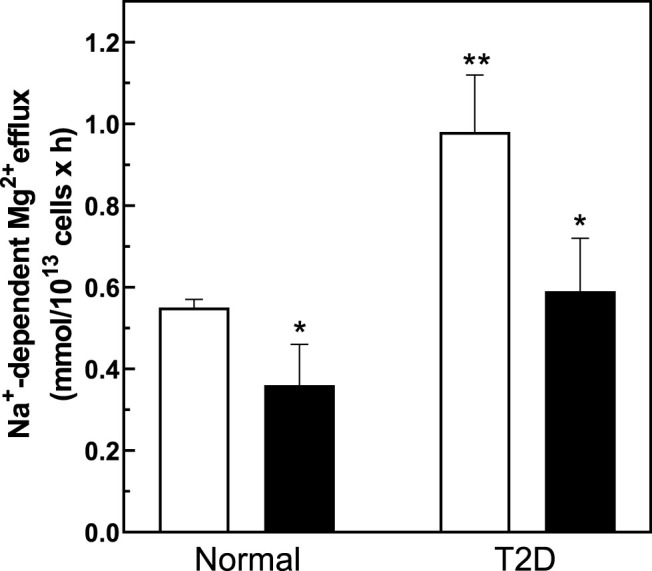
Regulation of the Na^+^/Mg^2+^ exchanger activity by PNGase treatment. Cells from normal and diabetic subjects were pre-incubated in the absence (white bar) or presence (black bars) of 0.1 U/mL PNGase F for 1.5 h and assayed for Na^+^/Mg^2+^ exchanger activity as described in *Methods*. Values are expressed as means ± SEM of three and five experiments in triplicate determinations in normal and diabetic subjects, respectively. Non-parametric paired *t* test of control vs. de-glycation in both normal (*p < 0.03) and diabetic subjects (*p < 0.001). Unpaired t test nonparametric of control in normal vs. control in diabetic subjects (**p < 0.0001).

To investigate if changes in cell surface N-linked protein glycosylation state might regulate Na^+^/Mg^2+^ exchange activity, we incubated intact RBC with peptidyl-N-Glycosidase F (PNGase F) to remove accessible N-linked glycans from RBC surface proteins (Watala, 1988; Tarentino and Plummer, 1994). PNGase F is an amidase that cleaves N-linked glycoproteins between the innermost GlcNAc and asparagine residues. In T-cells, pre-treatment with PNGase F *in vitro* reduced N-glycan content of cell surface proteins (Cabral et al., 2017) without significant loss of T cell regulatory function. To investigate if surface protein N-glycosylation contributes to Na^+^/Mg^2+^ exchange activity, we preincubated RBC with PNGase F following similar experimental protocols. We found that 1 h pretreatment of intact RBC from T2D subjects with PNGase F significantly reduced Mg^2+^ efflux to values comparable to those of normal RBC (Figure 6). This reduced Na^+^/Mg^2+^ exchange activity was also associated with a significant increase in cellular Mg content (*n* = 5, *p* < 0.05, Table 2). In RBC from healthy subjects, we further observed a decrease in Na^+^/Mg^2+^ exchanger activity in RBC from healthy subjects following PNGase F pretreatment of the intact cells, similarly associated with changes in intracellular Mg^2+^ (*n* = 3, *p* < 0.05, Table 2). These results are consistent with the possibility that ecto-N-glycosylation state of the Na^+^/Mg^2+^ exchanger polypeptide at the cell surface, or of an interacting regulatory protein, contributes to maintenance of erythrocyte Mg^2+^ homeostasis.

**TABLE 2:** Total cellular Mg^2+^ content in PNGase-treated red cells.

	Untreated cells	PNGase-treated cells	*p* values
Normal (*n* = 3)	2.0 ± 0.1	2.1 ± 0.06	<0.034
Diabetic (*n* = 5)	1.8 ± 0.1	2.1 ± 0.1	<0.031

Erythrocytes from normal and diabetic subjects were pre-incubated with with the N-glycanase (PNGase F), as described in Methods, and analyzed for total cellular Mg^2+^ as indicated. Values represent the means of triplicate determinations of three independent experiments. Mg^2+^ levels are expressed as mmol/Kg Hb. *p* values were determined by paired Wilcoxon text.

**FIGURE 7:**
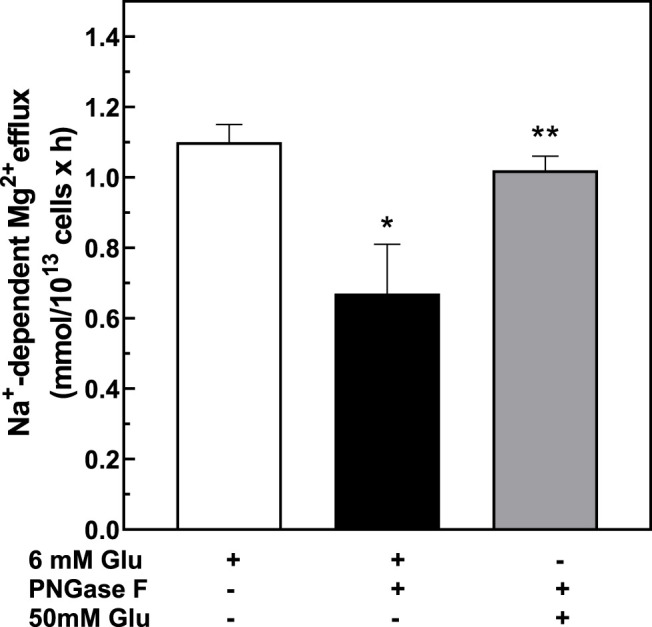
Effect of PNGase-treatment on glucose-stimulated exchanger activity. Cells from T2D subjects were treated with 6 mM glucose or 50 mM glucose for 1.5 h after pre-treatment with 0.1 U/mL PNGase F and assayed for Na^+^/Mg^2+^ exchanger activity as described in Methods. Values are expressed as means ± SEM of three experiments in triplicate determinations. * Non-parametric paired *t* test between 6 mM glucose (white bar) vs. PNGase-treated (black bar): *p < 0.002. Non-parametric paired t test between PNGase-treated vs. PNGase- and 50 mM glucose-treated (gray bar),**p < 0.05.

To assess reversibility of the effects of N-deglycosylation on Na^+^/Mg^2+^ exchange activity, we measured high glucose-induced Na^+^/Mg^2+^ exchange activity following PNGase F treatment. Interestingly, exposure of erythrocytes to high concentration of glucose after PNGase F treatment restored the higher levels of Na^+^/Mg^2+^ exchange to pre-PNGase levels (Figure 7). The reversibility of PNGase-F-induced inhibition of Na^+^/Mg^2+^ exchange by high extracellular glucose suggests that the stimulation of red cell Na^+^/Mg^2+^ exchange by high glucose, reflecting either red cell protein glycation or another effect of glucose, can override the inhibitory effect of protein ecto-N-deglycosylation on Na^+^/Mg^2+^ exchange. Thus, both RBC protein glycation state and RBC surface protein N-glycosylation state may contribute to RBC Mg^2+^ homeostasis through regulation of RBC Na^+^/Mg^2+^ exchange.

## Discussion

We hypothesized that glucose modulates intracellular ionic composition of RBC, and that dysregulated ion metabolism characterizes the pathophysiology of T2D. We report our observation that HbA_1c_ levels correlate directly with Na^+^/Mg^2+^ exchange activity and inversely with intracellular Mg levels in RBC from healthy subjects. The dysregulated erythroid Mg^2+^ homeostasis in T2D reflects enhanced glucose-regulated Na^+^/Mg^2+^ exchange activity. These findings suggest that hemoglobin glycation state predicts intracellular Mg levels controlled by regulation of Mg^2+^ efflux pathways in RBC, and complement previous observations of the inverse association between RBC Mg levels and fasting blood glucose (Resnick et al., 1993b; Barbagallo et al., 1996; Barbagallo et al., 2001). Na^+^/Mg^2+^ exchange thus joins the K_ATP_ channel, the L-type Ca^2+^ channel and GLUT4 of the pancreatic beta cell, K channel Kir4.1 of retina, and (*via* insulin) TRPM6 and the thiazide receptor NCC of kidney (Gommers et al., 2016) as a Mg^2+^-regulated ion transport pathway.

The regulation by glucose of Na^+^/Mg^2+^ exchange activity could in principle reflect the hyperglycemia-associated hypomagnesemia of diabetes, which has been attributed to diabetic glycosuria. Indeed, acute glucose infusion can promote magnesuria, and the SGLT2 knockout mouse exhibits hypermagnesemia. However, SGLT2 inhibitors used to treat T2DM do not promote magnesuria, but rather had been observed to lead to hypermagnesemia (Tang et al., 2016).

We also report pharmacological evidence that glucose-stimulated activation of Na^+^/Mg^2+^ exchange requires Src family tyrosine kinase activity. Inhibition of Src family tyrosine kinase activity was sufficient to attenuate the increased Na^+^/Mg^2+^ exchange activity or T2D RBC. Src tyrosine kinase functions in part as downstream signaling molecule for receptors without intrinsic kinase activity (Parsons and Parsons, 1997). While the mechanism by which RBC Src tyrosine kinase regulates Na^+^/Mg^2+^ exchange remains to be determined, Src kinase and caveolin are known together to contribute to activation of the receptor for advanced glycation end-products (RAGE) in vascular tissues (Reddy et al., 2006). RAGE mediates inflammatory and white blood cell migration-associated signals associated with vascular complications of hyperglycemia (Igarashi et al., 1999; Schmidt, 2015; Hudson and Lippman, 2018). Thus, the Src family of tyrosine kinases may be potential targets for development of new therapeutic strategies to ameliorate glucose-mediated complications associated with T2D.

Nonenzymatic glycation affects not only hemoglobin, but extends to proteins of the RBC surface (Miller et al., 1980). Red cell glycation status has been correlated with cellular survival and with rheology that is modifiable by control of glycemic status (Peterson et al., 1977). Hyperglycemia has also been associated with decreased red cell membrane fluidity (Watala, 1988). However, a direct relationship between red cell surface glycation status and transport protein function remains to be established. Nonetheless, exposure to extracellular glucose modulates activities of the Na^+^ pump (Garner et al., 1990; Umudum et al., 2002; Nandhini and Anuradha, 2003), Ca^2+^ pump (González Flecha et al., 1993), Na^+^/H^+^ exchanger (Williams and Howard, 1994), GLUT1 (Nandhini and Anuradha, 2003), as well as the non-erythroid Na^+^/Glucose cotransporter of epithelia (Han et al., 2005). We have extended these studies to demonstrate that glucose regulates erythroid Mg^2+^ levels by modulating Na^+^/Mg^2+^ exchange activity. Consistent with a role for deregulated cation metabolism in the pathogenesis of T2D, we found reduced cellular Mg^2+^ levels and increased Na^+^/Mg^2+^ exchanger activity in *ex vivo* RBC from T2D subjects as compared to cells from otherwise healthy subjects. *ex vivo* RBC from *db/db* mice showed Mg^2+^ transport abnormalities resembling those of RBC from T2D subjects. Exposure of RBC to extracellular glucose abolished apparent cooperativity in the extracellular Na^+^ -dependence of Na^+^/Mg^2+^ exchange activity, accompanied by reduction in K_1/2_ for extracellular Na^+^ from 83 to 29 mM. This change may reflect a noncovalent effect of glucose or a direct or indirect effect of non-enzymatic glycation at or near the putative extracellular Na^+^ binding site of the Na^+^/Mg^2+^ exchanger, or of residues in one or more of the exchanger’s hypothesized regulatory proteins.

Covalent reaction of the open form of glucose with proteins leads to protein glycation (Rabbani and Thornalley, 2012) occurs predominantly on N-terminal α-amino groups and ε-amino groups of Lys residues, as well as on side chains of Arg and Cys residues. Early glycation proceeds sequentially through glycosylamine formation and dehydration to Schiff bases in a process requiring hours. Subsequent Amadori rearrangement to fructosamine or non-Amadori rearrangement to α-oxo-aldehydes, both of which can then more slowly degrade over weeks *in vitro* (Valencia et al., 2004) to multiple advanced glycation end-products (AGEs). Protein susceptibility to glycation and the gradually increasing burden of protein glycation in circulation and in cells likely contributes to pathogenesis of hyperglycemic disorders (McAvan et al., 2020).

SLC41A1 has been often (Sponder et al., 2013), if controversially (Sahni and Scharenberg, 2013), modeled as 10 transmembrane spans with intracellular N- and C-termini. In this model, SLC41A1 has four ecto-Lys residues in two putative extracellular loops and multiple ecto-Arg residues in each of the five predicted extracellular loops, any of which might serve as glycation targets. As glucose-induced activation of Na^+^/Mg^2+^ exchange occurs within 1.5 h, the observed effects of extracellular glucose on RBC Na^+^/Mg^2+^ exchange activity do not likely reflect the action of AGE formation of binding to their receptors (RAGEs). The time- and concentration-dependence of Na^+^/Mg^2+^ exchange activation by extracellular glucose, is consistent with those previously reported by Watala et al. for red cell membrane glycation (Watala, 1988). Also consistent with our results, impaired Mg^2+^ uptake and homeostasis characterizes the streptozotocin-induced rat model of type 1 diabetes (T1D) (Cefaratti and Romani, 2003). Hepatic plasmalemmal vesicles from streptozotocin-treated rats, exhibited >2-fold higher rates of Na^+^-dependent Mg^2+^ efflux than those from untreated rats (Cefaratti et al., 2004; Fagan et al., 2004). These data together demonstrate that hyperglycemia, as observed in T2D and in a model of T1D, induces changes in Mg^2+^ transport.

We found that RBC pre-treatment with extracellular PNGase F restores Na^+^/Mg^2+^ exchange activity to normal levels, reversing its elevation by high glucose levels. PNGase F exposure at 37°C for 1 h has been used to partially remove surface N-linked glycans from intact T regulatory cells without compromising cellular function (Cabral et al., 2017). We showed that PNGase F treatment of RBC from T2D subjects increased glucose-mediated intracellular Mg levels and decreased Na^+^/Mg^2+^ exchanger activity, suggesting that enzymatic removal of RBC surface protein-linked N-glycans can regulate Mg^2+^ homeostasis in T2D RBC. The 10-transmembrane span model of SLC41A1 predicts a single consensus site for ecto-N-linked glycosylation, but neither mutational nor enzymatic evidence for this site has yet been presented. Thus, the functionally important RBC surface polypeptide substrate(s) for extracellular PNGase regulation of erythroid Na^+^/Mg^2+^ exchange remain to be defined.

Enhanced glycation of RBC and endothelial surface proteins can enhance vascular dysfunction, suggesting that attenuation of protein glycation might delay development of vascular complications in T2D. Our results suggest that RBC surface glycation and N-glycan state of the Na^+^/Mg^2+^ exchange protein or of one or more interacting regulatory protein(s) can regulate intracellular Mg^2+^ and Mg^2+^ homeostasis in T2D RBC. Additional work is needed to identify the glycated and glycosylated proteins responsible for Na^+^/Mg^2+^ exchange activation in RBC of T2D subjects. Also remaining to be investigated is the possible effect on Na^+^/Mg^2+^ exchange of the recently reported deposition of amylin aggregates in or on T2D red cells (Verma et al., 2020). As regulation of RBC Mg^2+^ transport may have parallels in endothelial and vascular smooth muscle cells, we speculate that identification and modulation of Mg-regulatory glycation and N-deglycosylation targets may have therapeutic potential in treatment of the vascular complications of T2D.

## Data Availability

The original contributions presented in the study are included in the article/Supplementary Material, further inquiries can be directed to the corresponding author.
